# Abstracts/Sommaires/Zusammenfassungen

**DOI:** 10.1155/S1463924679000717

**Published:** 1979


### Abstracts Sommaires Zusammenfassungen

### Page 249

### An experiment in education for automatic analysis

D. Betteridge

During July 1979 a one-week residential School on Automatic Analysis was held at Swansea. It consisted of lectures, small group discussions and practical classes. Being the first of its kind, it was an experiment in education for Automatic Analysis. The organisation of the school is described, and the longer term educational issues arising from it are discussed.

### Une expérience dans l'éducation de l'analyse automatique

En juillet 1979 un cours d'une semaine en analyse automatique a eu lieu à Swansea. Le cours consistait de conférences, de discussions en petits groupes et de travaux pratiques en petits groupes. Etant le premier cours dans son genre ce fut une expérience dans l'éducation de l'analyse automatique. L'organisation de l'école et du cours sont décrites et les issues à long terme sont discutées.

### Ein Experiment auf dem Gebiet der Ausbildung zur automatisierten Analytik

Im Juli 79 fand in Swansea ein einwochiger Ausbildungskurs zur automatisierten Analytik statt. Er bestand aus Vorlesungen und Diskussionen und praktischer Arbeit in kleinen Gruppen. Als erster Kurs dieser Art ist er ein Experiment auf dem Gebiet der Ausbildung zur automatisierten Analytik. Die Organisation des Kurses wird beschrieben und die daraus entstehenden längerfristigen Ausbildungsaspekte werden diskutiert.

### Page 252

### The development of an automated chemistry control system for secondary coolant circuits in CANDU Nuclear Power Reactors

J.R. Dean and R.B. Stewart

This paper summarizes the development of a method for automated control of the secondary coolant chemistry of CANada Deuterium Uranium 600 MW(e) power reactors using on-line analyzers, a minicomputer and chemical additon pumps. The development was carried out by the Analytical Science Branch of Whiteshell Nuclear Research Establishment (WNRE) of Atomic Energy of Canada Limited (AECL) in co-operation with Saskatchewan Power Corporation (SPC) at Estevan, Saskatchewan. Some results of a demonstration of the system are described.

### Développement d'un système de contrôle chimique automatique pour le circuit de refroidissement secondaire des réacteurs nucléaires CANDU

Ce travail résume le développement d'une méthode pour le contrôle automatique du circuit de refroidissement secondaire des réacteurs CANada Deuterium Uranium 600 MW(e) utilisant des analysateurs on-line, un minicomputer et des pompes pour ajout de réactifs. Le développement a été fait par la section analytique du Whiteshell Nuclear Research Establishment (WNRE) de la Atomic Energy of Canada Limited (AECL) en collaboration avec Saskatchewan Power Corporation (SPC) à Estevan, Saskatchewan. Quelques uns des résultats de la démonstration du système sont décrits.

### Die Entwicklung eines automatischen Chemie-Kontrollsystems fur Sekundär-Kühlkreise in CANDU Kernenergie-Reaktoren

Diese Arbeit ist eine Zusammenfassung der Entwicklung einer Methode für die automatisierte Steuerung der Sekundär-Kühlflüssigkeits-Chemie der CANada Deuterium Uranium 600 MW(e) Leistungsreaktoren, basierend auf der Verwendung von on-line Analysatoren, eines Minicomputers, und von chemischen Zudosierpumpen. Die Entwicklung wurde von der Analytical Branch of Whiteshell Nuclear Research Establishment (WNRE) der Atomic Energy of Canada Limited (AECL) in Zusammenarbeit mit der Saskatchewan Power Corporation (SPC) in Estevan, Saskatchewan, durchgeführt. Es wurden einige Resultate einer Demonstration des Systems beschrieben.

### Page 259

### Practical aspects of an on-line laboratory data system

A.P. Rowland and C.C. Blake

An on-line data processing system which collects and processes data from up to eight chemical instruments is described. Signals from atomic absorption spectrophotometers, continuous flow colorimeters and electronic balances are processed using system software and BASIC, before being collated into a suitable report form for customer output. Discussion of the operating procedures along with the advantages and disadvantages of the microprocessor based system is included.

### Aspects pratiques d'un système on-line de traitement de données de laboratoire

Un système on-line de traitement de données de laboratoire qui reçoit et traite les données de huit instruments chimiques au maximum est décrit. Les signaux de spectromètres par absorption atomique, de colorimètres à débit continu et de balances électroniques sont traités à l'aide du software du système, et en BASIC, pour être en suite comprimés dans un rapport pour le client. Les procédures opérationelles ainsi que les avantages et désavantages du système à microprocesseur sont décrits.

### Ein on-line Labordatensystem aus der Sicht der Praxis

Es wird ein on-line Datenverarbeitungssystem beschrieben, welches Daten von bis zu acht chemischen Instrumenten aufnimmt und verarbeitet. Signale von Atomabsorptions-Spektrometern, Durchfluss-Kolorimetern und elektronischen Waagen werden unter Verwendung von Systemsoftware und BASIC verarbeitet und nachher in geeigneter Form zu einem Bericht für den Kunden zusammengestellt. Betriebsablauf und Vor- und Nachteile des Mikroprozessor-Systems werden diskutiert.

Page 267

**An automatic monitor for lead emission from stacks: the design philosophy and primary evaluation**

C.J. Jackson

A device is described for the continuous automatic monitoring of emissions of lead dust and fume from stacks or ducts. Built by BNF Metals Technology Centre under contract to the Health and Safety Executive (HSE), the monitor isokinetically abstracts samples from the stack or duct, using a standard sampling probe and a Venturi-type injector, transmits the air-diluted samples to a remote spectrophotometer where lead atoms are produced by burning the samples in an air-propane flame. Excitation of these atoms, using the 283 nm emission line from a lead hollow cathode lamp, produces an atomic fluorescence signal which is detected and evaluated as g(lead) $M^{-3}$ (stack gas). Calibration and self-cleaning sequences are also incorporated. The construction and operation of the monitor is described as is its evaluation by both BNF and HSE. A number of operating problems discovered during these trials are discussed and possible solutions proposed.

**Un système automatique pour la surveillance de l'emission de plomb de cheminées: philosophie de construction et première évaluation**

Un instrument est décrit qui permet la surveillance automatique en continu des émissions de poussières et de fumées de plomb provenant de cuves et de canaux à air. Cet instrument de surveillance a été construit sur commande du Health and Safety Executive (HSE) par le BNF Metals Technology Centre. Il prend à l'aide d'un échantilleur standard et d'un injecteur Venturi, isocinétiquement, des échantillons des canaux à air, les transmet après dilution à l'air au spectrophotomètre éloigné, où des atomes de plomb sont produits dans une flamme air-propane. Les atomes sont excités à l'aide d'une lampe cathodique de plomb à vide à 283 nm et produisent des signaux de fluorescence atomique. Les signaux sont évalués et le résultat est donné en g (plomb) $m^{-3}$ (gaz de cheminée). Étalonnage et autonettoyage sont incorporés. La construction et le fonctionnement ainsi que l'évaluation par BNF et HSE sont décrites. Divers problèmes qui ont été rencontrés pendant la période d'essai sont discutés et des solutions sont proposées.

**Ein automatisches Ueberwachungsgerät für die Bleiemission von Kaminen: Konstruktionsphilosophie und erste Auswertung**

Es wird ein Gerät für die kontinuierliche automatische Ueberwachung der Emission von Bleistaub und Bleidämpfen von Luftschächten und Luftkanälen bechrieben. Das Ueberwachungsgerat wurde im Auftrag der Health and Safety Executive (HSE) von BNF Metals Technology Centre gebaut. Es entnimmt unter Verwendung eines Standard-Probennehmers und einem Venturi Einspritzgerät isokinetisch Proben aus dem Schacht oder Kanal und schickt die luftverdünnten Proben zu einem weiter entfernten Spektrophotometer, wo Bleiatome durch Verbrennung der Proben in einer Luft-Proben Flamme erzeugt werden. Die Anregung dieser Atome unter Benützung der 283 nm Emissionslinie einer Blei-Hohlkathodenlampe erzeugt atomare Fluoreszenzsignale, welche detektiert und zu g (Blei) $m^{-3}$ (Kamingas) ausgewertet werden. Eichung und Selbstreinigungsschritte sind ebenfalls eingebaut. Konstruktion und Betrieb des Monitors sind zusammen mit der Evaluation durch BNF und HSE beschrieben. Eine Reihe von Betriebsproblemen, sind diskutiert, die während dieser Versuchsphase aufgetreten sind, und es werden dazu Lösungen vorgeschlagen.

Page 273

**An evaluation of the Kodak Ektachem system for the determination of glucose and urea**

R. Haeckel

An investigation has been made of the analytical reliability of the Kodak Ektachem analytical system. The principles of the technique are described, and method and results for the determination of glucose and urea concentrations in human blood sera are given. The interference due to compounds added to the sample sera is assessed and an indication of the practicability of the system is given.

**Expériences avec le système Kodak Ektachem pour la détermination de glucose et d'urée**

La fiabilité du système analytique Kodak Ektachem a été etudié. Les principes de cette technique sont décrits, les méthodes et des résultats de la détermination de glucose et d'urée dans le serum de sang humain sont donnés. Les interférences dues à des substances ajoutées au echantillons de sérum sont évaluées et les possibilités d'application du système sont discutées.

**Erfahrungsbericht über die Glucose- und Harnstoff-Bestimmung mit dem Kodak Ektachem System**

Die Arbeit berichtet über eine Untersuchung der analytischen Zuverlässigkeit des Kodak Ektachem Analysensystems. Die Prinzipien der Technik werden beschrieben, und Methodik und Resultate fur die Bestimmung von Glucose und Harnstoff in menschlichem Blutserum sind angeführt. Die Interferenz durch Verbindungen, die der Serumprobe beigefügt wurden, werden ermessen und eine vorläufige Beurteilung der praktischen Nützlichkeit des Systems wurde abgegeben.

